# Development and Validation of an Enzyme-Linked Immunosorbent Assay-Based Protocol for Evaluation of Respiratory Syncytial Virus Vaccines

**DOI:** 10.3390/v16060952

**Published:** 2024-06-12

**Authors:** Eliel Nham, A-Yeung Jang, Hyun Jung Ji, Ki Bum Ahn, Joon-Yong Bae, Man-Seong Park, Jin Gu Yoon, Hye Seong, Ji Yun Noh, Hee Jin Cheong, Woo Joo Kim, Ho Seong Seo, Joon Young Song

**Affiliations:** 1 Division of Infectious Diseases, Department of Internal Medicine, Korea University College of Medicine, Seoul 02841, Republic of Korea; e.nham@kumc.or.kr (E.N.); grkmcgrid013@kumc.or.kr (A.-Y.J.); kormid@korea.ac.kr (J.G.Y.); hyeya44u@korea.ac.kr (H.S.); jynoh@korea.ac.kr (J.Y.N.); heejinmd@korea.ac.kr (H.J.C.); wjkim@korea.ac.kr (W.J.K.); 2 Vaccine Innovation Center-KU Medicine (VIC-K), Seoul 02841, Republic of Korea; harbe3103@korea.ac.kr (J.-Y.B.); ms0392@korea.ac.kr (M.-S.P.); 3 Korea Atomic Energy Research Institute, Jeongeup 56212, Republic of Korea; hyunjung@kaeri.re.kr (H.J.J.); ahnkb@kaeri.re.kr (K.B.A.); 4 Department of Microbiology, Korea University College of Medicine, Seoul 02841, Republic of Korea

**Keywords:** respiratory syncytial virus, RSV, ELISA, vaccine, immunogenicity

## Abstract

Recently, respiratory syncytial virus (RSV) vaccines based on the prefusion F (pre-F) antigen were approved in the United States. We aimed to develop an enzyme-linked immunosorbent assay (ELISA)-based protocol for the practical and large-scale evaluation of RSV vaccines. Two modified pre-F proteins (DS-Cav1 and SC-TM) were produced by genetic recombination and replication using an adenoviral vector. The protocol was established by optimizing the concentrations of the coating antigen (pre-F proteins), secondary antibodies, and blocking buffer. To validate the protocol, we examined its accuracy, precision, and specificity using serum samples from 150 participants across various age groups and the standard serum provided by the National Institute of Health. In the linear correlation analysis, coating concentrations of 5 and 2.5 μg/mL of DS-Cav1 and SC-TM showed high coefficients of determination (r > 0.90), respectively. Concentrations of secondary antibodies (alkaline phosphatase-conjugated anti-human immunoglobulin G, diluted 1:2000) and blocking reagents (5% skim milk/PBS-T) were optimized to minimize non-specific reactions. High accuracy was observed for DS-Cav1 (r = 0.90) and SC-TM (r = 0.86). Further, both antigens showed high precision (coefficient of variation < 15%). Inhibition ELISA revealed cross-reactivity of antibodies against DS-Cav1 and SC-TM, but not with the attachment (G) protein.

## 1. Introduction

Respiratory syncytial virus (RSV) is a major respiratory pathogen in young children, which imposes a greater disease burden than influenza in this age group [1]. Now, it is being recognized as an important pathogen in older adults and immunocompromised individuals as well, causing acute exacerbation of pre-existing cardiovascular diseases or severe pneumonia [2,3,4]. Despite its clinical significance, the treatment and prevention tools for RSV remain limited, unlike seasonal influenza for which effective antiviral drugs and vaccines are available. Therefore, the World Health Organization is committed to developing effective RSV vaccines by actively collaborating with pharmaceutical companies [5]. As of January 2023, 22 vaccine candidates have entered clinical trials, 6 of which are in phase 3 [6]. Because several vaccine candidates have recently been reported to have satisfactory safety profiles and efficacy, one or more RSV vaccines are expected to be approved soon.

Among surface antigens of RSV, the fusion (F) protein and attachment (G) protein comprise the major immunogens, with the F protein being the preferred target of vaccines and monoclonal antibodies [7]. The F protein exists in two forms: a prefusion (pre-F) form before fusion with the host cell membrane, and a post-fusion form. Despite being more immunogenic than the post-fusion form, pre-F had not been used as a vaccine target because of its structural instability, a characteristic that led to the failure of RSV vaccine development in the past [7,8]. In the last decade, the structure of the pre-F form was revealed and subsequently engineered to make it more stable, with DS-Cav1 and SC-TM being the most widely used pre-F protein-based vaccine candidates [9,10]. DS-Cav1, developed by the Vaccine Research Center (VRC/NIH), is the first-of-its-kind stabilized pre-F protein [9]. SC-TM is one of the second-generation stabilized RSV pre-F antigens that shows further improved stability [10].

RSV vaccine candidates are based on various platforms, making it necessary to establish a standardized immunogenicity test protocol applicable to all vaccine products. Additionally, it is desirable to examine the correlation between simple and scalable enzyme-linked immunosorbent assays (ELISA) and neutralization tests, a labor-intensive gold standard. Therefore, we developed and validated an indirect ELISA-based protocol using the two most used modified pre-F proteins, DS-Cav1 and SC-TM, and G protein.

## 2. Materials and Methods

### 2.1. Study Population

Anonymized blood samples were collected from a total of 150 healthy children and adults, stratified by age groups: under 5 years, 5–18 years, 19–49 years, 50–64 years, and aged 65 years and above, from February to November 2021. This study was approved by the Institutional Review Board (of Korea University Guro Hospital, IRB No. 2022GR0149), and the requirement for informed consent was waived.

### 2.2. Protein Construct Design, Expression, and Purification

To purify F protein, Adeno-X 293 cells (Takara Bio Inc., Shiga, Japan) were used. The Adeno-X 293 Cell Line was a low-passage, transformed human embryonic kidney cell line for adenovirus production and was grown in Dulbecco’s modified Eagle medium (Thermo Fisher Scientific, Waltham, MA, USA), supplemented with penicillin–streptomycin (Thermo Fisher Scientific) and 10% fetal bovine serum (FBS; Biowest, Nuaillé, France). Genes expressing the His-tagged RSV A2 F protein (Genbank NP_044596.1) were designed as previously reported and synthesized by Bionics (Seoul, Republic of Korea) [10,11]. All recombinant proteins were cloned into the ectodomain of the F protein with a C-terminal fold on the trimerization domain. The His-tagged SC-TM was designed by replacing the p27 sequence with a seven-amino acid GS-rich linker sequence and additionally replacing three amino acids (N67I, S215P, and D486N) [10]. The His-tagged DS-Cav1 protein, containing four mutations (S155C, S190F, V207L, and S290C), has been previously described [9]. Each gene was cloned into the pAdenoX vector (Takara), and the linearized vectors were transfected into Adeno-X 293 cells. Proteins were purified by Ni-NTA chromatography (Thermo Fisher Scientific), and further purified by Strep-Tactin Sepharose chromatography (Iba-Life Sciences, Göttingen, Germany).

A synthetic gene encoding the RSV A2 (Genbank NP_044595.1) G protein (secreted form; sG, amino acids 48 to 298) was cloned into pET28a in-frame with a C-terminal tandem 6-histidine. The non-glycosylated recombinant G proteins were expressed in *E. coli* BL21(DE3) and purified by Ni-NTA chromatography (Thermo Fisher Scientific). All these purified proteins contained tags because we did not take the step to remove them.

### 2.3. Development and Optimization of the ELISA-Based Protocol

The activity of the three antigens was confirmed by serial dilution and inhibition ELISA. Each antigen was fixed on the hydrophilic immunoplate, Maxibinding Plate (SPL, Pocheon, Republic of Korea), at a concentration of 0, 1, 2.5, 5, 7.5, or 10 mcg/mL, and immobilized overnight at 4 °C. Serum samples were placed in phosphate buffer solution (PBS; Welgene, Gyeongsan, Republic of Korea) with 0.05% Tween 20 to 1:100, 1:400, and 1:1600, and PBS was used as a negative control. The secondary antibody, alkaline phosphatase-conjugated anti-human immunoglobulin G (IgG; Southern Biotech, Birmingham, AL, USA), was diluted to 1:2000, 1:4000, 1:6000, and 1:8000. AP substrate reagent (1 mg/mL *p*-nitrophenyl phosphate in the diethanolamine substrate buffer) was used as a colorimetric substrate. The optical density was measured at 405 nm (OD_405_) and 690 nm (OD_690_) using a Spectramax 190 plate reader (Molecular Devices, San Jose, CA, USA). Antibody titers were estimated based on the values obtained by subtracting OD_690_ from OD_405_. OD (405–690 nm) was applied to the standardized curve-fit four-parameter logistic method (4-PL) to calculate antibody titers (ELISA units/mL, EU/mL) of each clinical sample. The respective constant values of the 4-PL equation: $f1=min+(max-min)/\{1+{\left(\frac{x}{EC50}\right)}^{hillslope}\}$, were determined using standardized serum provided by the National Institute of Health. Concentrations at which coating antigens and dilution factors of secondary antibodies exhibited (1) high optical density at high sample concentrations and (2) low optical density with PBS were chosen. IgG levels (ELISA units/mL) were determined for each sample using the 4-PL equation after applying the OD values. OD readings at 405–690 nm were used if the dilution factor was less than or equal to 2.0. The final value was obtained by multiplying the difference between the OD of the sample and the OD of the highest concentration of pooled sera by the dilution factor.

In addition, the following four blocking agents were examined to select one that minimized non-specific antigen–antibody reactions: PBS with 0.05% Tween 20 (PBS-T), 1% bovine serum albumin (BSA)/PBS-T, 10% fetal bovine serum/PBS-T, and 5% skim milk/PBS-T. All experiments were performed in duplicate and the average values were compared. The blocking agent that exhibited the lowest optical density and highest correlation coefficient (r) was chosen. All chemical reagents used in this study were purchased from Sigma-Aldrich (St. Louis, MO, USA).

### 2.4. Validation of the ELISA-Based Protocol

The accuracy, precision, and specificity of the established ELISA-based protocol were evaluated as follows:

1. Accuracy: Low, medium, and high titers of National Institutes of Health-provided reference serum were serially diluted 2-fold, from 1:200 to 1:6400. All experiments were performed in duplicate for each concentration. The average optical density values were plotted against the antiserum concentrations, and the r values were derived by correlating the calculated antibody titers against the three RSV antigens.
2. Precision: One sample was used from each age group. Five samples were diluted 2-fold, from 1:200 to 1:25,600. The experiment was performed six times repetitively, and the coefficient of variation was calculated for each sample concentration.
3. Specificity: All 150 samples were adsorbed with each of the 3 homologous/heterologous RSV antigens (10 μg/mL of DS-Cav1, SC-TM, and G protein) for an hour with gentle shaking (300 rpm) at RT, following which the antibody titers were measured. Post-adsorption antibody titers were compared with those without adsorption (pre-adsorption). Cross-reactivity was determined based on whether the antibody titers were significantly lower after adsorption. Because RSV infection is mainly prevalent in young children, and because antibody specificity might be affected by the difference in the duration of time elapsed since the last RSV infection among the age groups studied, we investigated whether there was a difference in the antibody specificity based on age.

### 2.5. Correlation of Antibody Titers Determined by ELISA with Neutralizing Antibody Titers

We evaluated the correlation between antibody titers determined by ELISA and neutralizing antibody titers measured using the viral reduction neutralization test (VRNT). The VRNT was conducted on 30 samples, with 6 samples from each age group, and performed as follows: Human epithelial-2 cells (HEp-2) were seeded in a 96-well plate and cultured for 24 h at 37 °C. Sera were diluted 1/10 and inactivated at 56 °C for 30 min. RSV-RFP (RSV A2-K-line19F from Professor Martin L Moore) virus was diluted in PBS to a multiplicity of infection (MOI) of 2.5. Then, 50 μL of diluted virus was mixed with 50 μL of serum dilution and incubated for 1 h at 37 °C. A total of 60 µL of each virus–serum mixture was aliquoted to their designated cell-containing wells and incubated for 1.5 h at 37 °C, after which the mixture was removed and incubated in 100 µL of 2% Dulbecco’s modified Eagle medium overlay solution for 24 h at 37 °C. Culture medium was removed from the 96-well culture plate and wells were washed with 100 µL of PBS-T before incubating with the virus–serum mixture. After 24 h, cells were fixed with 4% paraformaldehyde for 30 min and then rinsed with PBS-T (0.05% Tween-20) wash buffer. Nuclei were stained by treating with 50 µL of 6-diaminidino-2-phenylindole solution (1:50,000) for 5 min at 20 °C and then washed with 100 µL of PBS-T. A total of 50 µL of distilled water was added to each well before imaging the sample plates using the Operetta CLS system (PerkinElmer, Waltham, MA, USA). The neutralizing dose was determined by counting the number of infected and non-infected cells. The correlation between antibody titers against the three RSV antigens measured using our ELISA protocol and the 50% neutralization dose (VRNT50) was examined.

### 2.6. Statistical Analyses

All statistical analyses were performed using the Statistical Package for the Social Sciences (SPSS) version 22.0 (SPSS Korea; Seoul, Republic of Korea). For the correlation between the pre-F protein concentration and OD, r values > 0.90 in the Pearson correlation analyses were considered acceptable. For precision, test conditions were considered equivalent and reproducible at a coefficient of variation ≤ 20%.

## 3. Results

### 3.1. Purification of Prefusion F Proteins and G Protein

**Figure 1:**
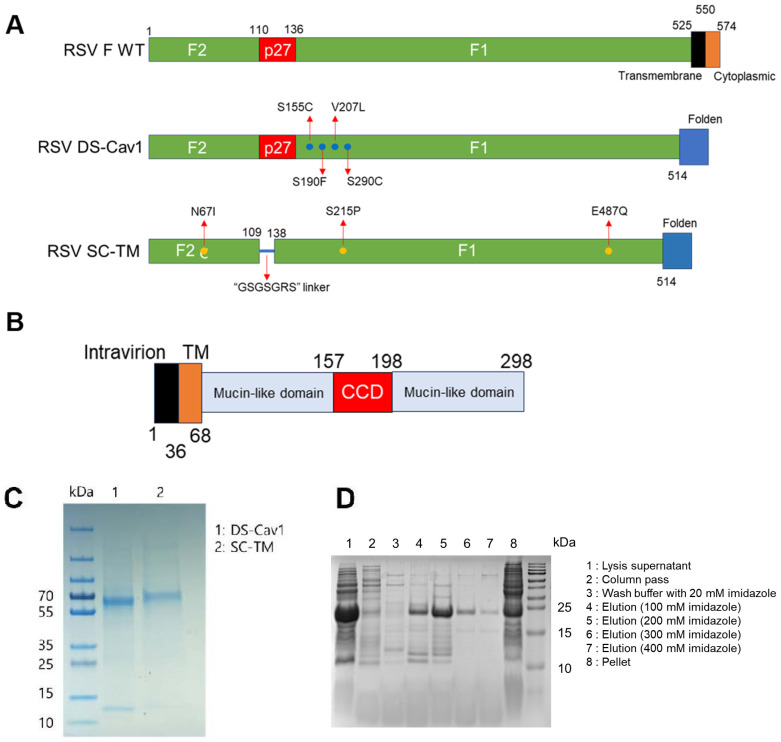
Purification of DS-Cav1, SC-TM, and G protein. (**A**) Schematic structure of RSV F antigen (Top), DS-Cav1 (Middle), and SC-TM (Bottom). (**B**) Schematic structure of RSV G protein. (**C**) SDS-PAGE of DS-Cav1 and SC-TM. (**D**) SDS-PAGE of recombinant G protein.

To validate RSV ELISA, we first purified F and G proteins, as described previously [9,10,12,13,14]. Two pre-F antigen vaccine candidates (DS-Cav1 and SC-TM) were expressed in human HEK293S cells and purified, while the G protein was overexpressed in *E. coli* BL21(DE3) and purified by Ni affinity chromatography. Four mutations (S155C, S190F, V207L, and S290C) were introduced to generate a stabilized pre-F protein (DS-Cav1), while a linker (GSGSGRS) between F1 and F2 and three mutations (N67I, S215P, and E487Q) were introduced to construct a chimeric stabilized pre-F protein (SC-TM; Figure 1A). The G protein was cloned from the extracellular region of the G protein, amino acids 68 to 298 (Figure 1B). After affinity chromatography and enrichment, DS-Cav1 was represented by two bands (F1 and F2) and SC-TM appeared on SDS-PAGE as one chimeric protein (Figure 1C). The G protein was approximately 25 kDa and was eluted from the Ni++ affinity column with increasing concentrations of imidazole (Figure 1D).

### 3.2. Development and Optimization of ELISA-Based Protocol

The concentration of DS-Cav1, as a coating antigen, was 5 μg/mL, and the dilution factor of the secondary antibody, at which a high optical density and r value greater than 0.9 was obtained, was 1:2000 (Table S1). The SC-TM and G proteins were coated at concentrations of 2.5 μg/mL and 5 μg/mL, respectively. The optimal dilution factor for the secondary antibody was 1:2000 and 1:6000 for SC-TM and G protein, respectively. For all three RSV antigens, 5% skim milk/PBS-T was used as the blocking reagent, as it exhibited the lowest optical density and an r value greater than 0.9, among the four blocking agents examined (Table S2).

### 3.3. Validation of the ELISA-Based Protocol

**Figure 2:**
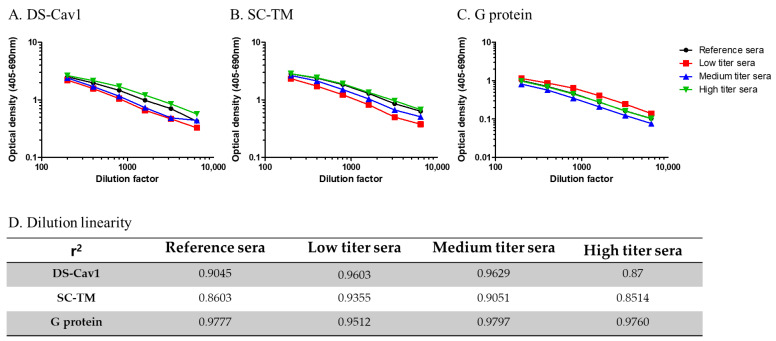
Accuracy (dilution linearity of optical density) of the respiratory syncytial virus enzyme-linked immunosorbent assay (RSV-ELISA). (**A**) DS-Cav1, (**B**) SC-TM, and (**C**) G protein. (**D**) Dilution linearity results with reference sera of the National Institutes of Health.

High accuracy was observed for all three RSV antigens. The value of r was greater than 0.85 for all antisera used. Overall, the r values measured with the reference antisera were 0.9045, 0.8603, and 0.9777 for DS-Cav1, SC-TM, and G protein, respectively (Figure 2 and Table S3). Anti-G protein antibody titers were higher for the low-titer reference sera compared to those for the medium and high reference sera, exactly the opposite from the anti-pre-F antibody titers (DS-Cav1 and SC-TM). The reference sera provided by the NIH were polyclonal sera categorized into high-, medium-, and low-titer sera based on their RSV-neutralizing antibody titers. While we were unable to identify any reports on anti-G protein antibody titers for those reference sera, our repeated experiments consistently demonstrated higher anti-G protein antibody titers in low-titer sera. Neutralizing activities are known to better correlate with pre-F antibody titers. There might be some discrepancies between anti-G protein antibody and neutralizing antibody titers.

As for the inter-assay precision, the coefficient of variation values were estimated to be below 15% for all RSV antigens and dilution factors. The mean coefficients of variation for DS-Cav1, SC-TM, and G protein were 2.708%, 2.664%, and 6.9115%, respectively (Table 1).

**Figure 3:**
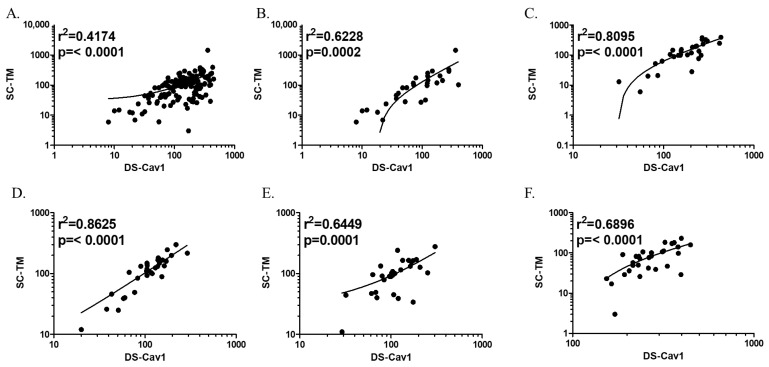
Correlation between anti-DS-Cav1 and anti-SC-TM immunoglobulin G antibodies (EU/L) by logistic regression. (**A**) Total population, (**B**) individuals aged < 5 years, (**C**) individuals aged 5–18 years, (**D**) individuals aged 19–49 years, (**E**) individuals aged 50–64 years, and (**F**) older adults aged ≥ 65 years. The dots and curves indicate antibody titers of study participants and their regression lines, respectively.

**Figure 4:**
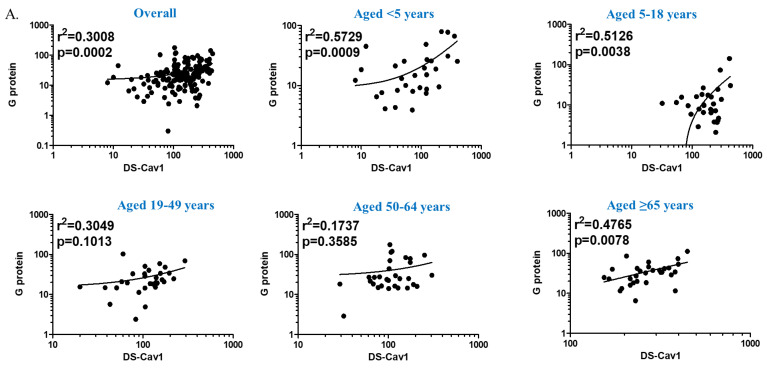

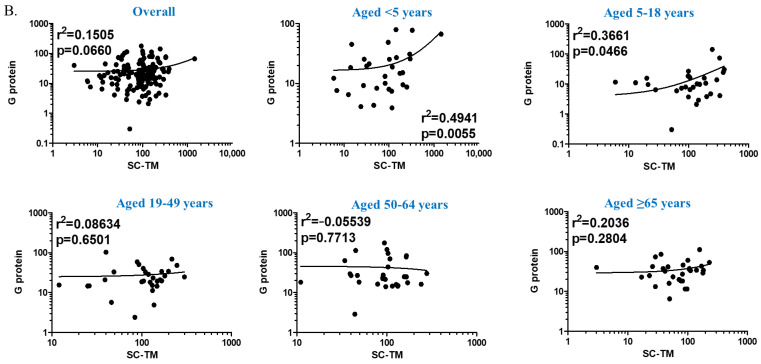
Correlation between anti-G protein and anti-prefusion F protein (anti-DS-Cav1 and anti-SC-TM) immunoglobulin G antibodies (EU/L) by logistic regression. (**A**) G protein versus DS-Cav1 and (**B**) G protein versus SC-TM. The dots and curves indicate antibody titers of study participants and their regression lines, respectively.

Two pre-F proteins (DS-Cav1 and SC-TM) exhibited high cross-reactivity (Figure 3). In comparison, pre-F and G proteins showed high specificity with each other, with low cross-reactivity (Figure 4). When adsorbed with a homologous antigen specific to each antibody, the anti-DS-Cav1, anti-SC-TM, and anti-G protein IgG antibody titers decreased to 49.6%, 42.6%, and 24.5%, respectively (Table 2). The DS-Cav1-specific antibody titers decreased by an average of 44.3% when adsorbed with the SC-TM protein. Similarly, SC-TM-specific antibody titers decreased by an average of 55.0% when adsorbed with the DS-Cav1 protein. Thus, antibodies against DS-Cav1 and SC-TM showed cross-reactivity with SC-TM and DS-Cav1 antigens, respectively. In comparison, both DS-Cav1- and SC-TM-specific antibody titers were reduced by less than 10% after adsorption with G protein.

The differences in antibody specificity by age groups were examined. The post-adsorption anti-DS-Cav1 IgG antibody titers decreased more significantly in individuals aged ≥ 65 years than in children aged < 5 years: anti-DS-Cav1 IgG antibody titers before/after adsorption with DS-Cav1, SC-TM, and G protein were 72.5%/22.6%, 68.0%/27.4%, and 106.9%/35.2%, respectively. Conversely, anti-SC-TM and anti-G protein antibody titers showed a greater decrease with adsorption in children aged < 5 years than in older adults aged ≥ 65 years. Anti-DS-Cav1 and anti-SC-TM IgG antibody titers were positively correlated (Figure 3). Although the r value derived from the entire study population was low (0.4174), the linear relationship became clearer when divided by age group and remained statistically significant. The correlation was strongest in individuals aged 19–49 years (r = 0.8625) and lowest in children aged < 5 years (r = 0.6228).

### 3.4. Correlation with Neutralizing Antibody Titers

**Figure 5:**
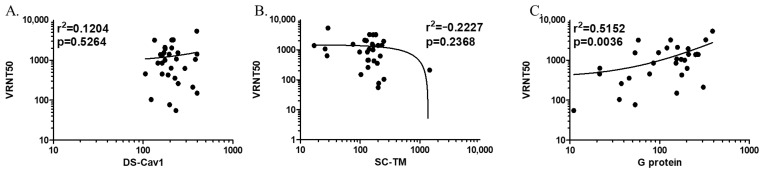
Correlation between anti-respiratory syncytial virus (RSV) protein immunoglobulin G (IgG) antibody titers (EU/L) and neutralizing activities by logistic regression. (**A**) Anti-DS-Cav1 IgG antibodies and neutralizing activities, (**B**) anti-SC-TM IgG antibodies and neutralizing activities, and (**C**) anti-G protein IgG antibodies and neutralizing activities. The dots and curves indicate antibody titers of study participants and their regression lines, respectively.

The correlation between anti-pre-F IgG antibody titers and neutralizing activities was weak. The value of r between IgG antibody titers and VRNT50 was 0.1204 (*p* = 0.526), and −0.2227 (*p* = 0.237) for DS-Cav1 and SC-TM, respectively (Figure 5). In contrast to the correlation between anti-pre-F antibodies and VRNT50, we found a positive correlation (r = 0.5152, *p* = 0.004) between anti-G protein IgG antibodies and VRNT50. When analyzed by age groups, the r values were estimated to be in the ranges of 0.1158–0.4795, 0.0309–0.1437, and 0.0042–0.6080 for DS-Cav1, SC-TM, and G protein, respectively.

## 4. Discussion

We established an ELISA protocol using two previously published, stabilized pre-F proteins (DS-Cav1 and SC-TM) and a non-glycosylated G protein, which showed good accuracy and precision. Antibodies against the two kinds of pre-F proteins showed remarkable cross-reactivity (as expected because the pre-F and post-F antigenic sites are preserved in these proteins). In comparison, anti-G protein antibodies were cross-reactive with neither anti-DS-Cav1 nor anti-SC-TM antibodies. There was poor correlation between anti-RSV protein IgG antibodies and neutralizing activities measured using the VRNT, indicating that those IgG antibodies had less- or non-neutralizing activities in the absence of vaccination.

When adsorbed with G protein, the SC-TM antibody titer was barely reduced (94.9% of the pre-adsorption) across all age groups; however, the DS-Cav1 antibody titer was considerably reduced by 37.2% in young children aged 5–18 years. Similarly, when adsorbed with pre-F proteins, the anti-G protein antibody titer decreased by approximately 50% on average in children. Young children are more likely to be repeatedly exposed to diverse respiratory viral antigens, which might result in non-specific antibody responses; therefore, the proportion of antibodies with lower specificity may be higher in children than in adults [15]. In children, antibody responses to RSV infection are influenced by independent factors, including the immature pediatric immune system and the presence or absence of maternal antibodies [16,17].

The low correlation between IgG pre-F antibody and neutralizing antibody titers might be due to the small amounts of functional antibodies. For this study, blood samples were collected from healthy individuals when RSV was in low circulation in the community because of the COVID-19 pandemic. A higher correlation would be expected when neutralizing antibody titers and post-vaccination RSV antibody are measured. Further studies are warranted to validate this assay in RSV vaccine recipients.

This study had some limitations. First, neither paired acute/convalescent sera nor postvaccination sera were available. Thus, specificity and correlation analyses were limited. Second, we did not test the neutralizing activity against the RSV B strain. However, the F protein genetic sequence is highly conserved between RSV strains, in contrast to the G protein, which shows approximately 50% genetic difference between RSV A and B strains [18]. Finally, the G protein produced in this study was not glycosylated due to the use of the *E. coli* expression system, making it difficult to compare what would actually happen in vivo. Nevertheless, Kawahara et al. reported that non-glycosylated G protein provided robust protection against RSV, as shown by the modest correlation with neutralizing activities in this study, particularly in children and adolescents (Figure 4) [19].

Since 2022, pre-F protein-based RSV vaccines with high efficacy in older adults and pregnant women have been reported [20,21,22,23]. A single dose of a protein-subunit vaccine or mRNA vaccine showed an efficacy of over 80% against RSV-related lower respiratory tract diseases in adults aged 60 years and above, with an acceptable safety profile during one RSV season [20,21,22]. The efficacy of the maternal immunization against medically attended, severe RSV-related lower respiratory tract disease in infants younger than 90 and 180 days of age has been reported to be 81.8% and 69.5%, respectively [23]. Pre-F is mainly used as the target antigen for RSV vaccines, but G protein, nucleocapsid, and matrix proteins are also targeted to enhance cell-mediated immunity [24]. The advent of RSV vaccines is welcome news at a time when RSV is rampant again after its transient disappearance at the onset of the COVID-19 pandemic [25,26,27]. Therefore, there is an urgent need to establish a standardized vaccine immunogenicity evaluation method. The ELISA protocol (based on two pre-F antigens and G proteins) developed and validated in this study may be helpful in evaluating vaccines that are to be introduced in the near future.

## Figures and Tables

**Table 1:** Inter-assay precision of the respiratory syncytial virus enzyme-linked immunosorbent assay (RSV-ELISA) for immunoglobulin G antibodies against prefusion F (DS-Cav-1 and SC-TM) and G proteins.

Antigen	DF *	Aged < 5 Years		Aged 5–18 Years		Aged 19–49 Years		Aged 50–64 Years		Aged ≥ 65 Years	
		EU/mL (Mean ± SD)	CV (%)	EU/mL (Mean ± SD)	CV (%)	EU/mL (Mean ± SD)	CV (%)	EU/mL (Mean ± SD)	CV (%)	EU/mL (Mean ± SD)	CV (%)
DS-Cav1	1:200	58.0 ± 0.6	0.99	67.0 ± 2.6	3.91	74.1 ± 2.1	2.89	30.0 ± 0.9	2.94	68.4 ± 2.0	2.88
	1:400	46.5 ± 1.0	2.15	73.5 ± 3.4	4.63	85.3 ± 2.3	2.69	31.0 ± 0.9	2.87	74.2 ± 3.6	4.84
	1:800	50.0 ± 1.2	2.31	89.9 ± 2.3	2.61	97.8 ± 2.2	2.20	40.4 ± 0.6	1.54	85.8 ± 2.9	3.40
	1:1600	70.8 ± 0.3	0.36	106.0 ± 3.1	2.95	115.7 ± 4.1	3.57	65.6 ± 0.4	0.56	112.3 ± 4.3	3.86
SC-TM	1:200	60.4 ± 1.3	2.10	81.7 ± 1.0	1.24	75.8 ± 3.4	4.54	40.4 ± 1.1	2.63	26.7 ± 0.8	2.94
	1:400	47.6 ± 2.5	5.28	104.0 ± 2.8	2.67	84.3 ± 3.4	4.00	40.4 ± 0.6	1.57	22.1 ± 0.7	2.97
	1:800	49.9 ± 0.9	1.71	132.1 ± 6.0	4.53	94.7 ± 4.7	4.95	45.3 ± 0.5	1.21	30.3 ± 0.3	1.05
	1:1600	62.8 ± 1.3	2.02	152.7 ± 4.1	2.69	112.8 ± 2.5	2.26	61.0 ± 1.2	2.02	50.0 ± 0.5	0.90
G protein	1:200	127.9 ± 7.3	5.72	16.1 ± 1.9	11.55	266.9 ± 17.1	6.40	163.3 ± 13.1	8.00	610.0 ± 22.0	3.60
	1:400	98.7 ± 4.5	4.51	15.9 ± 1.2	7.83	215.9 ± 15.6	7.24	117.9 ± 11.1	9.38	891.8 ± 51.1	5.73
	1:800	83.0 ± 4.1	4.99	24.2 ± 1.2	4.88	231.7 ± 14.3	6.19	112.5 ± 15.4	13.72	1224.9 ± 100.3	8.19
	1:1600	79.7 ± 1.9	2.43	45.2 ± 0.7	1.70	201.9 ± 10.8	5.37	104.4 ± 10.5	10.11	1572.2 ± 168.1	10.69

DF, dilution factor; EU/mL, ELISA units/mL; SD, standard deviation; CV, coefficient of variation. * All experimental results were multiplied by the dilution factor based on 100:1 and presented as antibody titer (EU).

**Table 2:** Specificity of immunoglobulin G antibodies against prefusion F (DS-Cav1 and SC-TM) and G proteins: comparison of antibody titers after adsorption with homologous and heterologous proteins.

Coated Protein		DS-Cav1				SC-TM				G Protein			
Inhibitor (10 μg/mL)		None	DS-Cav1	SC-TM	G Protein	None	DS-Cav1	SC-TM	G Protein	None	DS-Cav1	SC-TM	G Protein
Aged < 5 years	EU/mL	115.0	83.3	78.2	122.9	153.3	35.8	30.8	113.7	117.3	53.5	49.2	22.4
	%	-	72.5	68.0	106.9	-	23.3	20.1	74.2	-	45.6	42.0	19.1
Aged 5–18 years	EU/mL	193.8	73.0	67.3	121.6	148.4	69.3	65.7	122.5	78.8	44.1	41.9	17.3
	%	-	37.7	34.7	62.8	-	46.7	44.2	82.5	-	56.0	53.2	22.0
Aged 19–49 years	EU/mL	118.8	45.2	60.5	114.0	121.1	73.4	60.6	130.7	101.9	89.0	77.3	28.2
	%	-	38.1	51.0	96.0	-	60.6	50.0	108.0	-	87.4	75.9	27.6
Aged 50–64 years	EU/mL	122.7	65.7	84.0	107.7	107.0	88.0	61.1	120.4	134.3	145.8	132.1	42.3
	%	-	53.5	68.5	87.8	-	82.3	57.1	112.6	-	108.6	98.4	31.5
Aged ≥ 65 years	EU/mL	113.7	62.2	75.6	127.2	84.1	71.4	43.5	95.2	165.8	172.4	157.0	36.1
	%	-	54.7	66.5	111.9	-	84.8	51.8	113.2	-	104.0	94.7	21.8
Average	EU/mL	132.8	65.9	73.1	118.7	122.8	67.6	52.3	116.5	119.6	101.0	91.5	29.3
	%	-	49.6	55.1	89.4	-	55.0	42.6	94.9	-	84.4	76.5	24.5

EU, ELISA unit.

## Data Availability

The data presented in this study are available upon request from the corresponding author.

## Supplementary Materials

The following supporting information can be downloaded at: https://www.mdpi.com/article/10.3390/v16060952/s1. Table S1: Determination of coating antigen concentration and dilution factor of the secondary antibody. Table S2: Determination of optimal blocking buffer. Table S3: Accuracy (dilution linearity) of the respiratory syncytial virus enzyme-linked immunosorbent assay (RSV-ELISA) for immunoglobulin G antibodies against prefusion F (DS-Cav-1 and SC-TM) and G proteins.
