# Supplementary material for: Development and Validation of an Enzyme-Linked Immunosorbent Assay-Based Protocol for Evaluation of Respiratory Syncytial Virus Vaccines
Source: Viruses. 2024 Jun 12;16(6):952. doi: 10.3390/v16060952 (PMC11209066; doi:10.3390/v16060952)
Supplement: Supplementary file 1 [file viruses-16-00952-s001.zip › viruses-2984360-supplementary.pdf]

Table S1. Determination of coating antigen concentration and dilution factor of secondary antibody

			DS-Cav1 coating concentration (μg/mL)												Sample DF
			10		7.5		**5**		2.5		1		0		
AP-conjugated anti-human IgG DF	**1:2000**	1:6000	2.7741	1.7662	2.4809	1.6319	**2.4059**	1.7617	1.8144	1.3032	1.0369	0.7494	0.2278	0.2016	1:100
			1.6947	1.0093	1.4831	1.0247	**1.4225**	1.4176	1.0608	0.7942	0.5802	0.426	0.1092	0.1158	1:400
			0.7664	0.5221	0.6824	0.5021	**0.6734**	0.5106	0.479	0.3949	0.2729	0.2107	0.0818	0.0827	1:1600
			0.0715	0.0709	0.0747	0.0919	**0.0725**	0.0805	0.0708	0.6037	0.0708	0.0702	0.0697	0.0698	PBS
	**r^2**		0.9296	0.9440	0.9354	0.9268	**0.9371**	0.8453	0.9429	0.9512	0.9571	0.9582	0.9999	0.9950	
	1:4000	1:8000	1.6704	1.6284	1.4697	1.5678	1.4603	1.747	1.1704	1.4225	0.6914	0.7186	0.1547	0.1749	1:100
			1.0783	1.225	0.97	1.2021	1.1465	1.5979	0.7139	0.9101	0.3685	0.4341	0.0996	0.1086	1:400
			0.5191	0.6651	0.4609	0.6946	0.6474	1.5051	0.3454	0.4583	0.2097	0.2054	0.0791	0.0886	1:1600
			0.0708	0.0677	0.1356	0.151	0.3609	1.4337	0.0709	0.1775	0.0708	0.0775	0.0708	0.0791	PBS
	**r^2**		0.9163	0.8523	0.9216	0.8520	0.8830	0.9566	0.9358	0.9365	0.9682	0.9488	0.9956	0.9982	
			**SC-TM coating concentration (μg/mL)**												Sample DF
			10		7.5		5		**2.5**		1		0		
AP-conjugated anti-human IgG DF	**1:2000**	1:6000	2.8387	1.7967	2.7182	1.7786	2.652	1.8172	**2.126**	1.441	1.2594	1.0192	0.2436	0.2174	1:100
			1.7711	1.0943	1.6214	1.1105	1.5419	1.1354	**1.2207**	0.8784	0.6791	0.7013	0.1124	0.1047	1:400
			0.7899	0.5206	0.7126	0.4916	0.658	0.6246	**0.5444**	0.3997	0.3092	0.4742	0.0834	0.0806	1:1600
			0.0865	0.0796	0.071	0.0709	0.0718	0.2498	**0.072**	0.0698	0.0707	0.1721	0.0694	0.0693	PBS
	**r^2**		0.9256	0.9330	0.9362	0.9277	0.9428	0.9433	**0.9460**	0.9350	0.9619	0.9051	0.9998	0.9998	
	1:4000	1:8000	1.6484	1.6799	1.5593	1.601	1.5305	1.5315	1.3137	1.5005	0.7755	1.1824	0.1564	0.1628	1:100
			1.0881	1.3165	1.0463	1.2929	1.0517	1.298	0.8156	1.0907	0.4945	0.7146	0.0977	0.1049	1:400

			0.5102	0.6846	0.4741	0.7172	0.465	1.1113	0.3949	0.5752	0.2808	0.95	0.0779	0.0795	1:1600
			0.0798	0.0892	0.0779	0.1023	0.2775	0.4798	0.0709	0.0753	0.2124	0.0911	0.0706	0.0746	PBS
	**r^2**		0.9120	0.8404	0.9084	0.8218	0.9276	0.7626	0.9297	0.8716	0.9661	0.7038	0.9978	0.9945	
			**G protein coating concentration (μg/mL)**												Sample DF
			10		7.5		**5**		2.5		1		0 .		
AP-conjugated anti-human IgG DF	1:2000	**1:6000**	3.011	1.7195	2.8393	1.665	2.7137	**1.5454**	2.3192	1.3668	1.537	1.046	0.1698	0.1492	1:100
			2.0961	1.1712	1.7733	1.0795	1.5666	**0.9654**	1.1828	0.7629	0.6004	0.4409	0.064	0.0662	1:400
			1.1998	0.6661	0.9499	0.5395	0.7395	**0.4216**	0.4407	0.2835	0.2026	0.1471	0.0367	0.0381	1:1600
			0.7952	0.4199	0.5949	0.2027	0.2384	**0.1192**	0.1828	0.0603	0.0533	0.0348	0.0258	0.0271	PBS
	**r^2**		0.9389	0.9419	0.9595	0.9323	0.9548	**0.9356**	0.9746	0.9555	0.9927	0.9880	0.9999	0.9975	
	1:4000	1:8000	2.6963	1.6475	2.5196	1.5633	2.387	1.4626	2.1244	1.2552	1.3519	0.9052	0.1336	0.1971	1:100
			1.788	1.1832	1.5941	1.067	1.4305	0.9407	1.0904	0.776	0.5674	0.4214	0.0876	0.0579	1:400
			1.0323	0.7528	0.8727	0.6033	0.6792	0.4546	0.4265	0.3092	0.2166	0.1625	0.0374	0.0383	1:1600
			0.5684	0.4139	0.4338	0.3118	0.2048	0.1074	0.0812	0.0638	0.0426	0.0418	0.0256	0.0291	PBS
	**r^2**		0.9423	0.9193	0.9482	0.9307	0.9461	0.9245	0.9680	0.9343	0.9881	0.9810	0.9426	0.9967	

Abbreviations: AP, alkaline phosphatase; DF, dilution factor; PBS, phosphate buffered saline

Table S2. Determination of optimal blocking buffer

		DS-Cav1 (5 μg/ml)					
		**1:100**	**1:400**	**1:1600**	**1:6400**	**PBS**	**r²**
AP-conjugated anti-human IgG (1:2000)	PBS-T	2.1863	1.236	0.563	0.2178	0.0696	0.955
		2.1255	1.1619	0.5145	0.2109	0.0622	
	Average	2.1559	1.19895	0.53875	0.21435	0.0659	
	1% BSA/PBS-T	2.0293	1.0965	0.5054	0.1929	0.0607	0.9561
		1.9722	1.1182	0.483	0.2055	0.0617	
	Average	2.00075	1.10735	0.4942	0.1992	0.0612	
	10% FBS/PBS-T	1.9805	1.0823	0.4631	0.1889	0.064	0.9608
		1.9768	1.0511	0.472	0.1896	0.0625	
	Average	1.97865	1.0667	0.46755	0.18925	0.06325	
	5% skim milk/PBS-T	1.8606	0.9758	0.418	0.1763	0.0636	0.9623
		1.9056	1.0426	0.4648	0.1892	0.069	
	Average	1.8831	1.0092	0.4414	0.18275	0.0663	
		**SC-TM (2.5 μg/ml)**					
		**1:100**	**1:400**	**1:1600**	**1:6400**	**PBS**	**r²**
AP-conjugated anti-human IgG (1:2000)	PBS-T	1.6844	1.1055	0.507	0.1974	0.0654	0.9307
		1.7375	1.0485	0.4605	0.1842	0.0623	
	Average	1.71095	1.077	0.48375	0.1908	0.06385	
	1% BSA/PBS-T	1.6646	1.0178	0.4398	0.1877	0.0646	0.9445
		1.7225	0.9906	0.4502	0.1804	0.0604	

	Average	1.69355	1.0042	0.445	0.18405	0.0625	
	10% FBS/PBS-T	1.5759<br>1.6129	0.9836<br>0.9713	0.4138<br>0.435	0.1801<br>0.1773	0.0605<br>0.0599	0.938
	Average	1.5944	0.97745	0.4244	0.1787	0.0602	
	5% skim milk/PBS-T	1.5442<br>1.573	0.8907<br>0.9209	0.4027<br>0.4292	0.1719<br>0.1648	0.0618<br>0.0655	0.9482
	Average	1.5586	0.9058	0.41595	0.16835	0.06365	
		**G protein (5 μg/ml)**					
		**1:100**	**1:400**	**1:1600**	**1:6400**	**PBS**	**r^2**
AP-conjugated anti-human IgG (1:6000)	PBS-T	1.4734<br>1.4565	0.8668<br>0.803	0.374<br>0.3938	0.2265<br>0.2241	0.0557<br>0.1692	0.9975
	Average	1.46495	0.8349	0.3839	0.2253	0.11245	
	1% BSA/PBS-T	1.5394<br>1.5562	0.9335<br>0.9857	0.447<br>0.4639	0.2291<br>0.2246	0.1358<br>0.1224	0.9925
	Average	1.5478	0.9596	0.45545	0.22685	0.1291	
	10% FBS/PBS-T	1.435<br>1.5384	0.8117<br>0.8139	0.3355<br>0.3376	0.1328<br>0.1324	0.0493<br>0.0472	0.9975
	Average	1.4867	0.8128	0.33655	0.1326	0.04825	
	5% skim milk/PBS-T	1.4735<br>1.4966	0.8452<br>0.853	0.356<br>0.3469	0.1236<br>0.1226	0.0287<br>0.0285	0.9949
	Average	1.48505	0.8491	0.35145	0.1231	0.0286	

Abbreviations: AP, alkaline phosphatase; PBS, phosphate buffered saline; BSA, bovine serum albumin; FBS, fetal bovine serum

Table S3. Accuracy (dilution linearity) of the respiratory syncytial virus enzyme-linked immunosorbent assay (RSV-ELISA) for immunoglobulin G antibodies against prefusion F (DS-Cav-1 and SC-TM) and G proteins.

**DS-Cav1 (5 µg/ml)**	**Dilution factor**						r^2
	**1:200**	**1:400**	**1:800**	**1:1600**	**1:3200**	**1:6400**	
**Reference sera**	2.519	1.9658	1.4659	0.9846	0.7047	0.42405	0.9045
**Low titer sera**	2.2103	1.5678	1.0532	0.6568	0.46605	0.3265	0.9603
**Medium titer sera**	2.3991	1.70445	1.161	0.73195	0.4868	0.43985	0.9629
**High titer sera**	2.6452	2.1615	1.70685	1.21075	0.84935	0.56785	0.87
**SC-TM (2.5 µg/ml)**	**Dilution factor**						r^2
	**1:200**	**1:400**	**1:800**	**1:1600**	**1:3200**	**1:6400**	
**Reference sera**	2.83735	2.38555	1.8442	1.2892	0.85635	0.63475	0.8603
**Low titer sera**	2.34215	1.73725	1.2267	0.82085	0.4996	0.37665	0.9355
**Medium titer sera**	2.64995	2.1299	1.50995	1.037	0.66725	0.5086	0.9051
**High titer sera**	2.8413	2.41785	1.90155	1.34495	0.966	0.6745	0.8514
**G protein (5 µg/ml)**	**Dilution factor**						r^2
	**1:200**	**1:400**	**1:800**	**1:1600**	**1:3200**	**1:6400**	
**Reference sera**	1.01015	0.70585	0.4602	0.2764	0.16345	0.10335	0.9777
**Low titer sera**	1.14245	0.86765	0.6395	0.40675	0.2446	0.13945	0.9512
**Medium titer sera**	0.81455	0.5709	0.34935	0.2087	0.12385	0.0763	0.9797

**High titer sera**	0.97235	0.68775	0.44775	0.2775	0.1618	0.10215	0.9760

r^2, coefficient of determination
